# What is the prevalence of extra-articular and intra-articular magnetic resonance imaging findings in football players with and without hip and/or groin pain? A cross-sectional study of 166 football players

**DOI:** 10.1177/11207000261416847

**Published:** 2026-03-22

**Authors:** Emma Buckthorpe, Joshua J Heerey, Tom Entwistle, Kay M Crossley, Alex Davies, Matthew G King, Mark J Scholes, Joanne L Kemp

**Affiliations:** 1 School of Medicine, Monash University, Melbourne, Australia; 2 La Trobe Sport and Exercise Medicine Research Centre, School of Allied Health, Human Services and Sport, La Trobe University, Melbourne, Australia; 3 Imaging at Olympic Park, Melbourne, Australia; 4 Discipline of Physiotherapy, School of Allied Health, Human Services and Sport, La Trobe University, Melbourne Australia

**Keywords:** Athletes, football, hip, magnetic resonance imaging

## Abstract

**Aim:**

The primary aim of this study was to describe the prevalence of MRI findings in football players with and without hip and/or groin pain (HGP).

**Methods:**

We described the prevalence of extra-articular findings (including gluteal, hamstring, iliopsoas, rectus femoris; pubic) and intra-articular (including labral, cartilage, bone marrow oedema, subchondral and paralabral cysts, ligamentum teres) findings on MRI for 215 eligible hips with HGP (132 participants) and 68 eligible asymptomatic hips (34 participants). Imaging of the hip joint included radiographs and a non-contrast 3.0 Tesla MRI, performed at a single imaging centre.

**Results:**

There was no difference between groups for prevalence of any extra-articular or intra-articular findings. Pubic (75–85%), labral (70–78%) and chondral (54–60%) findings were most common. ⅓ of hips demonstrated all 3 findings regardless of symptoms (33.5% in HGP group and 35.5% in control group). 96.7% of HGP participants and 95.6% of control participants had at least 1 finding.

**Conclusions:**

Our study found that prevalence of extra- and intra-articular hip MRI findings did not differ between football players with and without HGP. Football players with and without pain were likely to have multiple imaging findings in 1 hip. A thorough history and clinical examination should be used to contextualise any imaging findings.

## Introduction

Hip and/or groin pain (HGP) is common in active young to middle-aged populations, with >½ of patients with longstanding groin pain presenting to a sports medicine practice being diagnosed with hip pathology.^1,2^ Amongst football players, approximately 50% will experience some form of HGP during a season.^3^ Such injuries also account for up to 18% of football-related time loss injuries,^2,4^ and is the ⅓ most common injury in the Australian Football League.^5^ The source of pain can be challenging to diagnose due to the abundance of potential pathologies in the region and the commonly seen multifactorial nature of the pain.^6,7^

Imaging is frequently used to assist with diagnosis and management of HGP, with the assumption that structural changes relate to symptom severity.^8^ Yet, there is increasing recognition that pain-free hips may also demonstrate structural changes or “abnormal” imaging findings.^9–11^ For instance, >60% of aymptomatic football players exhibit magnetic resonance imaging (MRI)-defined labral tears and adductor tendon pathology.^11,12^ Currently, there is little understanding of the overlap between imaging-defined intra- and extra-articular pathology and if specific groupings of conditions are related to hip and groin symptoms. Understanding this link may improve the appropriateness of assessment and ultimately treatment of athletes with hip and groin pain who often present with multiple potential sources of pain.

The primary aim of this study was to describe the prevalence of MRI findings in football players with and without HGP. The secondary aim was, in football players with and without HGP, to explore the overlap between (1) intra- and extra-articular pathologies; and (2) the 3 most common findings identified in aim 1.

## Methods

### Ethics and consent

This study was approved by the La Trobe University Human Ethics Committee (HEC 15-019 and HEC 16-045) and La Trobe Human Ethics Committee (2015000916 and 2016001694). All participants completed written, informed consent prior to commencing the study.

### Study participants

This study used baseline data collected from the Femoroacetabular impingement and hip OsteoaRthritis Cohort (FORCe) study, a longitudinal cohort study of football players evaluating changes in hip joint structure over time.^13^ Participants were recruited who fulfilled the study eligibility criteria in Table 1. Briefly, we recruited male or female football players (Australian Football or soccer) with HGP for >6 months and a positive flexion-adduction-internal rotation (FADIR) test, and a control group of football players without HGP. A group of asymptomatic football players was also recruited. Most importantly, asymptomatic football players were required to report no previous hip or groin injury or current HGP. All participants (HGP and control) were recruited using social media, printed advertisements, and information sessions at sporting organisations between August 2015 and October 2018. For this present study, we included Melbourne-based participants from the larger FORCe cohort study, as they were evaluated for extra-articular conditions using a standardised clinical assessment procedure.

**Table 1.** Eligibility criteria.

Inclusion Criteria	Exclusion Criteria
**Both groups**	
• Aged 18 to 50 years old • Playing in a sub-elite Australian football or soccer competition in Victoria, Australia, and undertaking at least 2 sessions (games or training) per week • Able to undergo imaging assessment at radiology clinic in Melbourne, Australia	• Any lumbar spine or lower limb injury/complaint in the previous 3 months that would prevent full weightbearing or ability to undergo full testing procedures • Previous hip and/or pelvis surgery • Contra-indications to radiography or MRI • Kellgren and Lawrence grade 2 OA changes or greater on AP pelvis radiograph • Unable to understand spoken and written English
Hip and/or groin pain group	
• 6 months of hip and/or groin pain of gradual onset • Pain with football or football related movements; sitting, squatting, kicking or cutting/change of direction+/- Symptoms including clicking, giving way, locking or catching • Positive FADIR test in at least one hip	• Self-reported history of significant hip or groin conditions; specifically: bursitis, congenital dislocation of the hip, fractures, osteochondritis dissecans, Legg-Calvé-Perthes disease, septic or rheumatoid arthritis, slipped capital femoral epiphysis or subluxations/dislocations • Received intra-articular hip injection (of any type in the previous 3 months
Asymptomatic group	
• Negative FADIR test in both hips	• Self-reported history of hip and/or groin pain, or significant hip or groin condition • Past history of any lower limb surgery including hip and/or pelvis surgery

AP, anterioposterior; LCEA, lateral centre-edge angle; MRI, magnetic resonance imaging; FADIR, flexion/adduction/internal rotation test.

### Participant assessment

Data for the present study were collected during the baseline assessment for the larger longitudinal FORCe study. Participants’ demographics included age, sex, sport played (Australian football or soccer), height and mass. Self-reported HGP symptom severity was measured using the International Hip Outcome Tool (iHOT-33), which has appropriate psychometric properties for use in this population.^14^

### Radiology methods

Each participant underwent 2 forms of imaging of the hip joint, an anteroposterior (AP) pelvis and Dunn 45° radiograph and a non-contrast 3.0 Tesla MRI. All imaging was completed at 1 imaging centre in Melbourne (Imaging @ Olympic Park, Melbourne, Australia). Radiographs were taken using a standardised protocol, as reported previously.^15^

For the MRI, the right and left hips were imaged separately with a 32-channel torso coil over the hips and pelvis. Participants were positioned in supine with each hip maintained in internal rotation and neutral abduction/adduction with patient positioning aids. The MRI sequences acquired were: coronal proton density (PD) spectral attenuated inversion recovery (SPAIR) (repetition time [TR] = 2700 ms, echo time [TE] = 25 ms, slice thickness [ST] = 2.5 mm, field of view [FOV] = 17 cm × 17 cm); sagittal PD (TR = 2095 ms, TE = 20 ms, ST = 2.0 mm, FOV = 17 cm × 17 cm), sagittal PD SPAIR (TR = 2675 ms, TE = 25 ms, ST = 2.5 mm, FOV = 15 cm × 15 cm) and oblique axial PD SPAIR (TR = 3500 ms, TE = 25 ms, ST = 2.5 mm, FOV = 17 cm × 17 cm and an Axial Gre Dixon utilized IP,OP,W and F signal ( TR = 3.65 TE = 1.1 ST 3 mm and 1.5 mm slice gap FOV 270 cm × 27 cm ).

For each participant, radiographs and MRI sequences were reviewed concurrently by 1 blinded assessor (TE), a senior radiologist. All sequences were used to assess the extra-articular findings. The PD SPAIR sequences were used for musculoskeletal pathology and the axial Dixon sequence was used to assess intrapelvic pathology.

Extra-articular findings were reported as present or absent using a standardised radiological clinical assessment. Findings included tendon and muscular findings in the gluteal, hamstring, iliopsoas, and rectus femoris muscles. These changes were indicated by key words including, but not limited to, tendonitis, oedema, inflammatory change, cysts, fraying, or general changes. Other findings that were recorded included pubic findings, trochanteric bursitis, ischiofemoral impingement, femoral avascular necrosis (AVN) and ischial tuberosity or femoral stress response.

Intra-articular findings were graded by one blinded musculoskeletal radiologist at the University of California San Francisco using the Scoring of Hip Osteoarthritis with MRI (SHOMRI) scoring system which has been described in detail previously.^16^ The SHOMRI scoring system evaluates 8 different findings of hip OA including: articular cartilage; bone marrow oedema pattern (BMEP); subchondral cysts; labrum; paralabral cysts; intra-articular bodies; effusion-synovitis; and ligamentum teres. An articular cartilage defect was scored as present if either partial or full thickness cartilage loss was evident in at least 1 acetabular (anterior, posterior, superomedial and superolateral) or femoral (anterior, posterior, superomedial, superolateral, lateral and inferior) subregion. A labral tear was scored as present if a grade 2 or above was evident in at least 1 of the 4 acetabular subregions.

A senior medical student (EB) who was trained to extract the relevant data by the radiologist (TE) extracted findings from the reports. They were blinded to the participants group, sport and BMI although age and gender were known by the nature of the report software.

### Inter-rater reliability

MRI scans for 37 participants (74 hips) were used to assess agreement for the extra-articular findings. The reports of the experienced radiologist (TE) who reported all findings were compared to those of 6 other radiologists to determine agreement. Each radiologist remained blinded to the other reports. Cohen’s Kappa and prevalence-adjusted and bias-adjusted kappa (PABAK) score were then calculated. The level of agreement was defined as: ‘poor’ <0.00, ‘slight’ 0.00–0.20, ‘fair’ 0.21–0.40, ‘moderate’ 0.41–0.60, ‘substantial’ 0.61–0.80 and ‘almost perfect’ 0.81–1.00.^17^ The reliability of the intra-articular findings has been reported previously.^11^

### Data management

For football players who reported bilateral HGP that met the inclusion criteria, both hips were included in analyses, and only 1 hip was entered in the analyses for football players who had unilateral HGP. For asymptomatic football players, both hips were included in analyses.

### Statistical analysis

Data were assessed for normality using boxplots and Shapiro-Wilk analyses. Mann-Whitney and chi-squared tests were used to determine differences for demographics between football players with HGP and asymptomatic football players.

For the first aim, the per-hip prevalence of the MRI findings for football players with HGP and asymptomatic football players were described as percentages. Participants were considered on a per-hip analysis whereby the pain group had only their painful hip(s) included in the analysis and the control group had two pain-free hips included.

For aim 2, we presented the intersection of findings using Venn-diagrams separately for the HGP group and the control group. We reported prevalence of intra-articular and extra-articular findings per hip. This was done by sorting participants into 4 categories; intra-articular findings only, extra-articular findings only, both intra and extra-articular findings or neither. Separate analyses were conducted to contrast the pain and control groups. The intersection between the top 3 findings by prevalence (labral tears, cartilage defects and pubic findings) was also reported. The prevalence and overlap of findings were displayed separately for the pain and control groups. Statistical analyses were conducted in SPSS version 27 (SPSS Inc, Chicago, Illinois, USA) with alpha set to 0.05 for all analyses.

## Results

### Participants

In total, 603 football players with HGP responded to advertising or attended football screening services. Of these, 184 were included in the final study cohort. The control group had 147 respondents with 51 fulfilling the eligibility criteria and without missing data to be included in the FORCe study.

As only those participants based in Melbourne and imaged at the 1 radiology service were included, the study cohort was reduced to 132 HGP participants and 34 pain-free participants. The baseline demographics of these two groups are shown in Table 2. Of these 132 people (264 hips), 49 single hips were excluded as they did not fulfill the inclusion criteria, which left 215 hips from this group to be evaluated.

**Table 2.** Demographics characterisitic of football players with and without HGP.

Demographic data		
	HGP Group (*n* = 132, 215 hips)	Control Group (*n* = 34, 68 hips)
Sex (% female)	28 (21%)	12 (35%)
Age (Mean [SD]) - years	26.4 (5.8)	26.8 (4.1)
Sport	62% Australian football	64% Australian football
BMI (Mean [SD]) - kg/m^2^	24.53 (2.94)	24.23 (3.34)
iHOT-33 score (mean [SD])	64 (20)	98 (6)

HGP, hip/groin pain group; SD, standard deviation; BMI, body mass index; iHOT-33, international hip outcome tool.

### Inter-rater agreement

The findings for inter-rater agreement for the extra-articular findings are contained in the supplemental Table 1. The level of prevalence-adjusted and bias-adjusted kappa agreement ranged from slight (0.11 [-0.12–0.35]) to perfect (1.0 [1.0–1.0]). Agreement for the reporting of intra-articular findings has been reported previously.^11^

The prevalence of extra-articular and intra-articular findings in the symptomatic and asymptomatic groups are contained in Table 3.

**Table 3.** Prevalence of extra-articular and intra-articular findings in the HGP and control groups (per hip).

Characteristic	HGP group (number (%)) (*n* = 215)	Control groups (number (%)) (*n* =68)
Gluteal pathology	60 (27.9%)	19 (27.9%)
Hamstring tendon	20 (9.3%)	3 (4.4%)
Iliopsoas tendon	43 (20%)	9 (13%)
Rectus femoris tendon	14 (6.5%)	5 (7.4%)
Pubic findings	161 (74.9%)	58 (85.3%)
Ischiofemoral impingement	4 (1.9%)	2 (2.9%)
Ischial tuberosity stress	10 (4.7%)	5 (7.4%)
Femoral stress response	2 (0.9%)	0 (0%)
Enchondroma	5 (2.3%)	0 (0%)
Labral tears	150 (69.8%)	53 (77.9%)
Cartilage defects	115 (53.5%)	41 (60.3%)
Bone marrow oedema	12 (5.6%)	4 (5.9%)
Subchondral cysts	16 (7.4%)	11 (16.2%)
Paralabral cysts	50 (23.3%)	17 (25.0%)
Ligamentum teres	5 (2.3%)	3 (4.4%)
Loose body	2 (0.9%)	0 (0%)
Joint effusion	62 (28.8%)	15 (22.1%)

HGP, hip/groin pain group.

### Intersection of extra and intra articular findings

**Figure 1.**
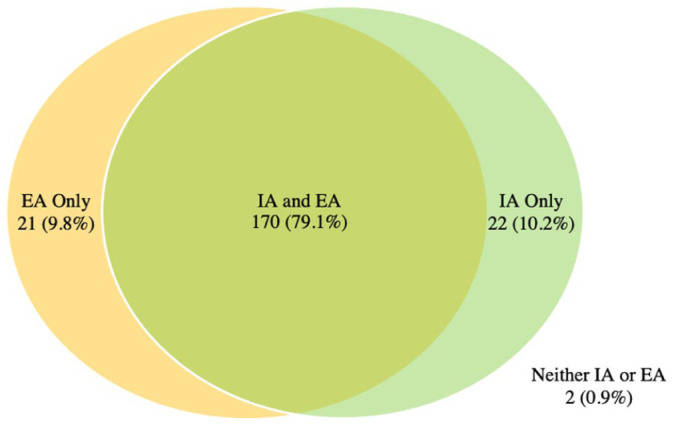
HGP Group: Intersection of intra-articular (IA) and extra-articular (EA) findings.

**Figure 2.**
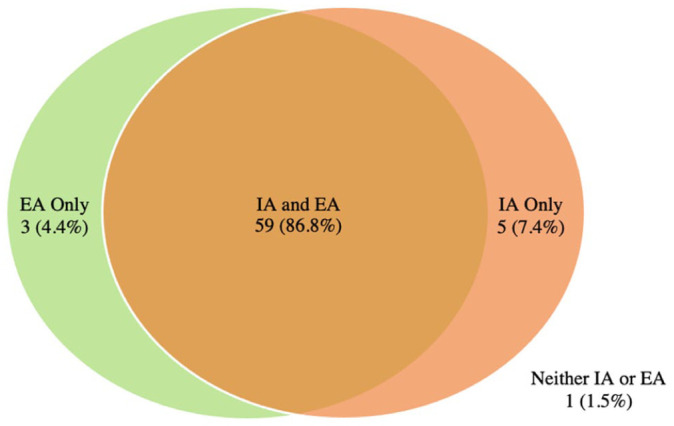
Control Group: Intersection of intra-articular (IA) and extra-articular (EA) findings.

Figures 1 and 2 demonstrate the prevalence of extra and intra-articular findings for football players with and without HGP, respectively. In both groups it was most common to have a hip with both an intra and extra-articular finding (86.8% and 79.1% respectively).

**Figure 3.**
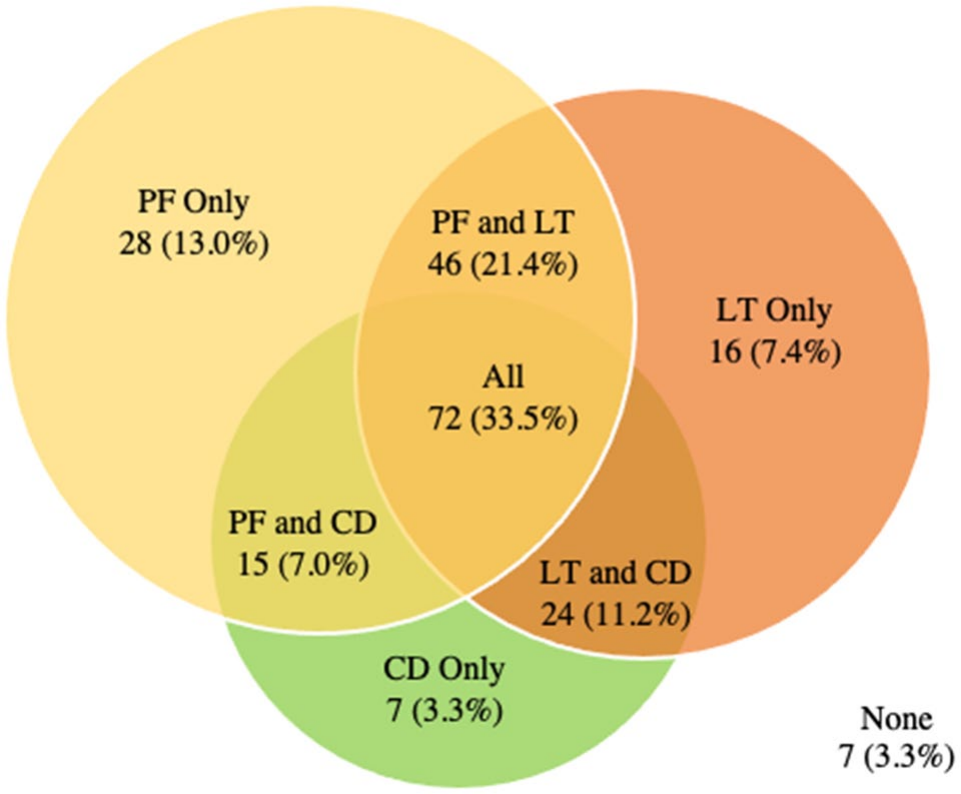
HGP Group: Intersection of the 3 most prevalent findings (pubic findings [PF], labral tear [LT] and chondral defect [CD]).

Figure 3 shows the intersection of the 3 most common pathologies amongst participants, these being labral tears, pubic findings and chondral defects. Within the HGP group, 96.7% of hips have at least one of the 3 most common findings and over a third (33.5%) have all 3 findings on imaging.

**Figure 4.**
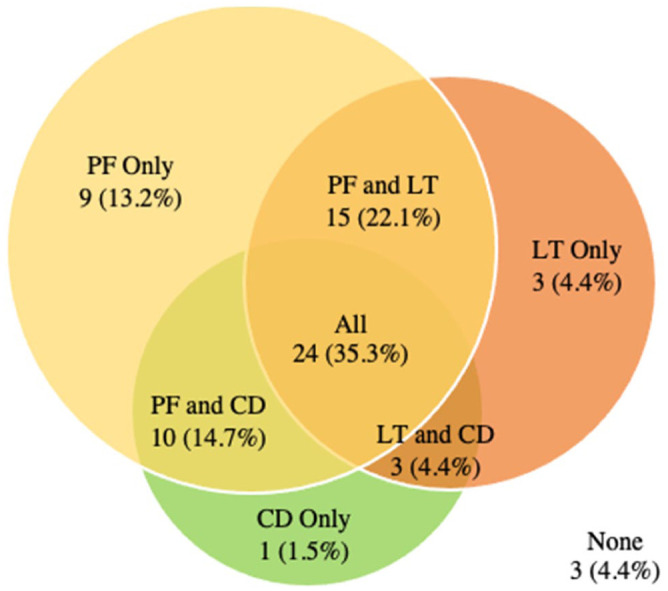
Control Group: Intersection of the 3 most prevalent findings (pubic findings [PF], labral tear [LT] and chondral defect [CD]).

Almost all (95.6%) of control hips also had at least 1 of the 3 most common findings and it was most common for hips to have all 3 findings (35.3%) (Figure 4). Labral tears were the most common finding with ⅔ (45 hips/66.2%) of control hips demonstrating this finding. Of these 45 hips; 42 had at least 1 of the other 2 findings.

## Discussion

Our study demonstrated that in football players, both extra-articular and intra-articular hip and pelvic imaging findings are frequently present independent of the presence of pain. The prevalence of all findings between HGP and asymptomatic football players were similar. Of note, the control group had no current or previously documented pain or injury on either hip, yet 98.5% of control hips had either an intra- and/or an extra-articular finding. There was also substantial overlap of the 3 most prevalent findings (labral tears, pubic findings, chondral defect) in both the control and HGP group. Over a third of hips demonstrated all 3 findings regardless of symptoms (35.3% of control hips and 33.5% of symptomatic hips).

The Doha agreement states that the likelihood of co-existing pathologies was high in athletes with HGP.^6^ We found the 3 most common findings in both participant groups were pubic findings, labral tears and chondral defects with prevalences of 85.3, 77.9% and 60.3% respectively in the control group and 74.9%, 69.8% and 53.5% respectively, within the HGP group. We have previously reported the high prevalence of cam morphology in this cohort of football players at 71% in those with HGP and 63% in those without.^15^ A recent systematic review indicates the prevalence of labral tears in symptomatic individuals is similar (62% [95% confidence interval (CI) 47–75%]) to those without symptoms (54% [95% CI, 41–66%]).^10^ Previous studies have shown a relationship between cam morphology, labral tears, chondral defects. Cam morphology size has also been shown to increase widening at the pubic symphysis during positions of impingement.^18^ Therefore, it is possible that there may be a relationship between these co-existing findings that has not yet been fully elucidated, although cause and effect is not clear. We also need to consider that these structures may not the source of HGP in football players. Imaging can identify structural changes but does not explore other physical contributors, nor the psychological or social contributors to pain. The severity of imaging findings of osteoarthritis of knee are not always related to the severity of pain or disability.^19^ Likewise, the high prevalence of severe degenerative spinal changes in asymptomatic people and low predictive value of many of these signs in low back pain,^20^ suggests that radiological changes may not mean a patient will have pain and, similarly, that pain may not be explained by radiological changes. Our research suggests the same might be true in athletes with HGP.

Our findings reinforce the notion that imaging should not be used without confirmatory clinical examination in football players with gradual onset HGP, as incidental MRI-defined extra-articular hip and pelvic conditions are present in a high number of asymptomatic football players. These findings have ramifications for clinical management and reinforces the notion that a patient’s pain, rather than isolated imaging findings, should be the primary guide for management. A thorough history and clinical examination should be used to contextualise any imaging findings and imaging is best used to either confirm a likely diagnosis or where clinical examination does not yield a reasonable diagnosis explore the possibilities of red flags. On its own, imaging is not adequate to determine the source of HGP. This is supported by previous research,^21^ which highlighted that MRI has limited additional utility over a thorough clinical examination and should be used judiciously.

The risk of overdiagnosis must be considered where findings in asymptomatic individuals could be labelled as pathology. This may lead to overtreatment, where clinicians may feel compelled to treat imaging findings, patients expect findings to be treated, and psychological barriers of feeling ‘injured’.^22^ This is likely to be not beneficial for patients with possible iatrogenic harm, but also places an unnecessary burden to the healthcare system.^22^ When patients are informed of findings on imaging, especially in medical jargon, it may change behaviour.^23^ When told there is a ‘finding’ or ‘pathology’, patients may not understand these terms,^24^ including their clinical significance and are often inclined to believe that they have some damage and therefore engage in activity modification or avoidance to prevent further damage and pain.^23^ Education programmes for athletes with HGP must discuss the uncertainty around the relationship between pain and structure, to ensure that athletes do not become fearful that movement and exercise will create further damage and subsequent pain to extra-articular and intra-articular hip structures.

Our study has several limitations. The reliability of reporting of findings was variable. This reflects variation seen in MRI reporting across a range of musculoskeletal conditions. Our control group was smaller than the symptomatic group, which may have led to the relative prevalence of findings being overstated when expressed as a percentage. There was also a significant difference in sex compositions of the 2 groups, where we had more men than women in both groups, reflecting the overall higher number of men than woman participating in community football. Given previous work, and references to the relationship between imaging findings and biomechanics in the discussion, sex-specific analyses ideally would have been undertaken. We did not conduct a standardised physical assessment of groin pain entities to substantiate imaging findings (for example, adductor tendon findings might be very relevant in people who also have a painful squeeze test [i.e., adductor-related pain]). Future studies should include a physical examination that explores the groin pain clinical entities.^6^

## Conclusion

Our study reported that prevalence of extra- and intra-articular hip MRI findings was not different between football players with and those without HGP. We also demonstrated that players were likely to have multiple findings in 1 hip: most frequently a combination of both extra- and intra-articular findings. The 3 findings with the highest relative prevalence (pubic findings, labral tears, and cartilage defects) frequently co-existed, and it was common for a hip, whether symptomatic or otherwise, to have all 3 findings.

## Supplemental Material

sj-docx-1-hpi-10.1177_11207000261416847 – Supplemental material for What is the prevalence of extra-articular and intra-articular magnetic resonance imaging findings in football players with and without hip and/or groin pain? A cross-sectional study of 166 football players Supplemental material, sj-docx-1-hpi-10.1177_11207000261416847 for What is the prevalence of extra-articular and intra-articular magnetic resonance imaging findings in football players with and without hip and/or groin pain? A cross-sectional study of 166 football players by Emma Buckthorpe, Joshua J Heerey, Tom Entwistle, Kay M Crossley, Alex Davies, Matthew G King, Mark J Scholes and Joanne L Kemp in HIP International
