# Supplementary material for: What is the prevalence of extra-articular and intra-articular magnetic resonance imaging findings in football players with and without hip and/or groin pain? A cross-sectional study of 166 football players
Source: Hip Int. 2026 Mar 22;36(3):486–92. doi: 10.1177/11207000261416847 (PMC13198615; doi:10.1177/11207000261416847)
Supplement: sj-docx-1-hpi-10.1177_11207000261416847 – Supplemental material for What is the prevalence of extra-articular and intra-articular magnetic resonance imaging findings in football players with and without hip and/or groin pain? A cross-sectional study of 166 football players [file sj-docx-1-hpi-10.1177_11207000261416847.docx]

**Supplemental Table 1.** Reliability for extra-articular findings.

| **Percent agreement, Cohen’s Kappa and PABAK for extra-articular findings** | | | |
| --- | --- | --- | --- |
| **Inter-observer agreement** | **Rate of agreement** | **Cohen’s Kappa (95% CI)** | **PABAK (95% CI)** |
| Gluteal pathology | 63/74 | 0.295 | 0.70 (0.54–0.86) |
| Iliopsoas | 40/74 | 0.057 | 0.08 (-0.15–0.86) |
| Hamstring | 54/74 |  | 0.46 (0.32–0.62) |
| Rectus femoris tendon | 63/74 | 0.307 | 0.70 (0.54–0.86) |
| Ischiofemoral impingement | 69/73 | -0.021 | 0.89 (0.79–0.99) |
| Public findings | 39/72 | 0.173 | 0.11 (-0.12–0.35) |
| Femoral stress | 74/74 | 1.000 | 1.0 (1.0–1.0) |
| Ischial Tuberosity Stress | 72/74 | 0.000 | 0.94 (0.87–1.02) |
| Enchondroma | 70/74 | 0.305 | 0.89 (0.79–0.99) |
